# Filopodia and Membrane Blebs Drive Efficient Matrix Invasion of Macrophages Transformed by the Intracellular Parasite *Theileria annulata*

**DOI:** 10.1371/journal.pone.0075577

**Published:** 2013-09-24

**Authors:** Min Ma, Martin Baumgartner

**Affiliations:** Division of Neuro-Oncology, Experimental Infectious Diseases and Cancer Research, Oncology Department, University Children’s Hospital Zürich, Zürich, Switzerland; University of Birmingham, United Kingdom

## Abstract

Recent technical advances have broadened our understanding of processes that govern mammalian cell migration in health and disease but many of the molecular and morphological alterations that precede and accompany movement of cells – in particular in three-dimensional (3D) environments - are still incompletely understood. In this manuscript, using high-resolution and time-lapse microscopy imaging approaches, we describe morphodynamic processes during rounded/amoeboid cell invasion and molecules associated with the cellular invasion structures. We used macrophages infected with the intracellular protozoan parasite *Theileria annulata*, which causes Tropical Theileriosis in susceptible ruminants such as domestic cattle. *T* *. annulata* transforms its host cell that, as a result, acquires many characteristics of human cancer cells including a markedly increased potential to migrate, disseminate and expand in the body of the host animal. Hence, virulence of the disease is associated with the capability of infected cells to disseminate inside the host. Using *T* *. annulata* -transformed macrophages as a model system, we described a novel mode of rounded/amoeboid macrophage migration. We show that filopodia-like membrane extensions at the leading edge lead the way and further evolve in blebbing membrane protrusions to promote progressive expansion of the matrix. Associated with focal invasion structures we detected ezrin, radixin, moesin-family proteins and their regulatory kinase MAP4K4. Furthermore, we linked Rho-kinase activity to contractile force generation, which is essential for infected cell motility. Thus, the motility mode of these parasite-transformed macrophages contrasts with those described so far in human macrophages such as the tunneling or mesenchymal modes, which require engulfment, compaction and ingestion of matrix or proteolytic matrix degradation, respectively. Together, our data reveal protrusion dynamics at the leading edge of invading cells in 3D at unprecedented temporal and spatial resolution and suggest a novel mode of rounded/amoeboid invasive cell motility that exploits actin-driven filopodia formation in combination with pressure-driven membrane blebs.

## Introduction

The capability to migrate is inherent to most eukaryotic cells and relevant under physiological or pathophysiological conditions such as inflammatory disorders and cancer. Cell motility is tightly regulated and motile cells can display plasticity of migration and adapt to environmental changes by modulating their mode of migration. Plasticity of cell migration is particularly relevant in three-dimensional (3D) environments where cells encounter not only a range of chemical cues but also mechanical constrictions and physical barriers. Based on morphological characteristics, cell motility in 3D is categorized in single cell motility and collective/cohort cell migration, whereby these two categories can be further subdivided into rounded/amoeboid or mesenchymal and multicellular streaming or sheet-like, respectively [1]. Plasticity of cell migration, although needed for normal physiological processes such as embryonic development, wound healing or immune response, is challenging treatment options for metastatic cancer. For example protease inhibitors for metastasis inhibition may fail because cancer cells can switch from mesenchymal (protease-dependent) to rounded/amoeboid (protease-independent) invasive motility modes, which resemble those of leukocytes migrating in interstitial tissues [2,3]. Importantly, motility at the cellular level is regulated by the interplay between the cell and the local chemical and biophysical environment. This interplay triggers still incompletely understood modifications in the cytoskeleton that control quality and extent of dynamic morphological alterations needed for cell propulsion [4,5]. Thus, a better understanding of the dynamic morphological alterations that drive invasive cell motility will improve our understanding of how cells control dissemination behavior in health and of how cancer cells can disseminate and metastasize.

To study invasive, rounded/amoeboid cell migration *in vitro*, we have chosen macrophages infected with the intracellular apicomplexan parasite *Theileria annulata*. This tick-transmitted parasite of ruminants can transform its host macrophages through chronic de-regulation of host cell signaling pathways. Still incompletely understood host cell transformation processes promote uncontrolled proliferation, long-term survival and parasite dissemination in the host animal [6]. Infected cells can be used as a reversible model of oncogenic transformation [7-12] because the parasite can be eliminated by parasitocidic treatment with the drug Buparvaquone 720c; hence transformation-dependent alterations can be determined and pathways that promote these alterations identified. Macrophages are a highly diverse set professional migratory cells that are present in almost all tissues to clear microorganisms, to initiate and mediate immune responses and to contribute to tissue repair [13]. Motility of macrophages is promoted by cytokines and chemokines, whereby the mode of macrophage migration in 3D is heavily influenced by the architecture of the extracellular matrix (ECM) [14]. *T* *. annulata* exploits macrophage versatility and triggers macrophage motile behavior to facilitate its dissemination in the host animal [12,15-17]. Parasite virulence and the underlying motile and invasive capability of infected cells are dependent on host and parasite factors. Specifically, infected host cells of susceptible animals produce increased levels of TGFβ in a parasite-dependent manner, which in turn triggers an rounded/amoeboid invasive motility program in the host cell through the activation of Rho kinase ROCK [10]. An analogous program downstream of TGFβ was described in human breast cancer cells that triggers dissemination of single cancer cells [18]. Consistently, protease-independent invasion of breast cancer cells *in vivo* is ROCK- and myosin-dependent [19], indicating the potential clinical relevance of approaches that target amoeboid/rounded cell invasiveness.

*T* *. annulata* -infected B cells [12] and macrophages [10] can penetrate gelled matrices derived from tumor cell ECMs (matrigel) *in vitro*, which is used in the cancer field to evaluate invasive capabilities of cells [20]. Little is known about the motility mode of *T* *. annulata* -infected or normal macrophages migrating in 3D matrices. However, two recent studies investigated human macrophage migration in 3D and the impact of matrix composition. These studies revealed that blood monocyte-derived macrophages switch form amoeboid to mesenchymal motility when the stiffness of the ECM increases [14]. Interestingly, these macrophages migrating in 3D can form adhesion and invasion structures that resemble 2D podosomes to facilitate matrix degradation and transmigration [21], which suggest that molecular mechanism driving local invasion structures in macrophages are conserved under different environmental conditions. In general, cells migrating in 3D environments can form a variety of matrix invading protrusions, including actin polymerization-driven lamellipodia- and filopodia-like protrusions or contractility-driven membrane blebs. Thus, eukaryotic cells can dynamically control what type of protrusion is generated because lamellipodia or blebs are formed independent of the overall cell morphology; accordingly it was suggested that protrusion formation is an autonomous module in the regulatory network that controls the plasticity of cell migration [4].

We recently demonstrated that the presence of intracellular *T* *. annulata* causes asymmetric activation of host cell actin dynamics, the induction of podosomes and the formation of a persistent lamellipodia in 2D [7]. However, the mode of cell motility of macrophages infected with *T* *. annulata* in 3D matrigel has not yet been investigated and what the analogous structures of podosomes and lamellipodia are in infected macrophages migrating in 3D is not known. In light of the recent conceptual progresses mentioned above, we searched to understand how *Theileria*-infected cells can penetrate matrigel matrices with such efficacy and to describe the necessary morphological and functional alterations. We chose to use macrophages infected with *T* *. annulata* , which were recently isolated [22] from Holstein cattle susceptible for tropical Theileriosis because they display an aggressive invasive behavior *in vitro* [10], which correlates with more virulent disease progression *in vivo*. Using these cells as a model system for rounded/amoeboid matrix invasion, we described a novel mode of invasive cell motility that involves the extension of filopodia-like membrane protrusions at the leading edge and subsequent matrix expansion by membrane blebs

## Materials and Methods

### Cell culture and reagents

TaH12810 cells (line H7, generous gift from Elizabeth Glass, The Roslin Institute, Edinburgh) were established *ex vivo* from the peripheral blood from Holstein calves previously infected with *T* *. annnulata* Hisar sporozoites [22]. The invasive and motile behavior of the TaH12810 cells has been recently described in more detail [10]. TaH12810 and Thei cells [23,24] (generous gift from Gordon Langsley) were cultivated in RPMI 1640 (Lonza) supplemented with 10% foetal calf serum (FCS, Amimed), 10 mM Hepes pH 7.2 (Merck), 2 mM L-glutamine (Gibco), 70 µM β-mercaptoethanol (Merck), and antibiotics (Lonza). Buparvaquone was a gift of Dirk Dobbelaere (Vetsuisse Faculty, Bern). TaH12810 cells expressing EGFP-actin or lifeact-mCherry (LA-mCherry) were generated by transfection with either pEGFP-hbeta-actin (generous gift of D. Gerlich; Institute of Molecular Biotechnology, Vienna) or pLenti-LA-mCherry (generous gift of Olivier Pertz). Plasmids: pEzrin-YFP [25] (generous gift of Miguel Quintavilla), moesin-GFP [26] (generous gift of Francisco Sánchez-Madrid). Chemicals: PP2 and SU6656 (Biaffin GmbH), H-1152 (Alexis Biochemicals, #ALX-270-423), Antibodies: mouse mc anti-MAP4K4 (clone MO7, Abnova), mouse mc anti-phospho Tyrosine (p-Tyr-100, 9411 Cell Signaling Technology), rabbit pc anti-Phospho-Ezrin (Thr567)/Radixin (Thr564)/Moesin (Thr558) (ERM) Antibody (Cell Signaling Technology, #3141). Rabbit pc anti-Ezrin/Radixin/Moesin (Cell Signaling Technology, #3142) and rabbit pc anti-TaSP [27] (generous gift from Jabbar Ahmed); TRITC/ALexa488 phalloidin (Molecular Probes).

### IF microscopy

Matrix-embedded cells were fixed in 4% paraformaldehyde solution in PBS for 15 min. Cells were then permeabilized by incubation in 0.5% TritonX-100 for 10 min. Blocking of non-specific epitopes was performed in blocking buffer (10% FBS in PBS, 0.02% sodium azide) for 15 minutes. Primary antibodies were applied at 1:50 to 1:200 dilutions in blocking buffer at 4°C over night. Non-confocal images were acquired in wide-field mode either on a Nikon 80i or on an inverted Nikon Eclipse TE2000-U microscope by using Openlab software. Confocal IF images were acquired on a laser-scanning microscope (Leica SP-2 and SP-5) using Leica software. Image procession and quantification were performed using Imaris, Adobe Photoshop and ImageJ software.

### Time-lapse imaging

Time-lapse imaging using video microscopy was performed using a Nikon Eclipse TE2000-U or a Leica LX inverted microscope, both equipped with a temperature- and CO2-controlled chamber. Data acquisition and image processing were performed using NIS software for the Nikon Instrument and Leica application suite (LAS) and ImageJ software for the Leica microscope. DIC and fluorescence images were acquired and assembled in AVI movies by NIS and exported for web in m4v format by QuickTime player.

### Matrix embedding

Cells were embedded in matrices in 15 well ibidi slides as described below: Collagen gels were prepared according to [28]. For 100 µl collagen solution, 5 µl sodium bicarbonate (7.5% stock, 50 mM final) was combined with 10 µl 10x PBS. 85 µl bovine collagen I solution (PureCol, 3 mg/ml stock, advanced BioMatrix) was added and carefully mixed on ice. 15 µl cell suspension (1-5 x 10^5^ cells/ml, 1.5-7.5 x 10^3^ cells/well) were mixed with collagen solution, transferred into the ibidi slides and allowed to polymerize for 30 minutes at 37°C in the incubator. This results in a final collagen concentration of 2.5%. Matrigel: 10 µl cell suspension at 1.5 x 10^5^ cells/ml were mixed with 90 µl growth factor-reduce basement membrane matrix (BD Biosciences), transferred into 15 well ibidi slides and allowed to polymerize for 30 minutes. OQel: 1 vial freeze-dried QGel matrix was resuspended in 500 µl ice-cold matrix resuspension buffer according to the manufacturers (QGel SA, Lausanne) instructions. 5 µl cell suspension at 3 x 10^5^ cells/ml were added to ready-made QGel matrix, transferred into ibidi slides and allowed to polymerize for 30 minutes at 37°C in the incubator.

### Rho-GTPase pull-down

Rho, Rac, Cdc42 pull-down assay (Cell Biolabs. Inc.) according to manufacturer instructions. In brief: Cells were lysed in pull-down lysis buffer (25 mM HEPES (pH 7.5), 150 mM NaCl, 1% NP-40, 1 mM EDTA, 2% glycerol). 800 µg protein was used per pull-down. Rho was pulled down using 40 µl slurry Rhotekin RBD agarose (20 µg bound Rhotekin RBD protein), and Rac and Cdc42 were pulled down using 40 µl slurry PAK1 PBD agarose (20 µg bound PAK1 PBD) for 1 h at 4°C. Pull-downs were then separated using 15% SDS-PAGE. Detection antibodies used: mouse mc anti-RhoA (No 240302), mouse mc anti-Rac1 (No 240106), mouse mc anti-Cdc42 (No 240201)

### Quantifications, repetitions and statistical analyses

Speed equals distance (d)/time (t) and the speed of single cells were determined as the average d/t_i,_ where t_i_ is the interval time. Bleb area was determined by measuring the area in µm^2^ of the bleb using ImageJ software.

Quantitative data for speed and motility mode determination were collected from at least three independent experiments. IFA studies and Rho-GTPase pull downs were done at least twice.

T-tests (unpaired, two-tailed) for statistical analysis were performed using Prism software.

## Results

### Rounded/amoeboid motility of infected macrophages in collagen and matrigel

**Figure 1:**
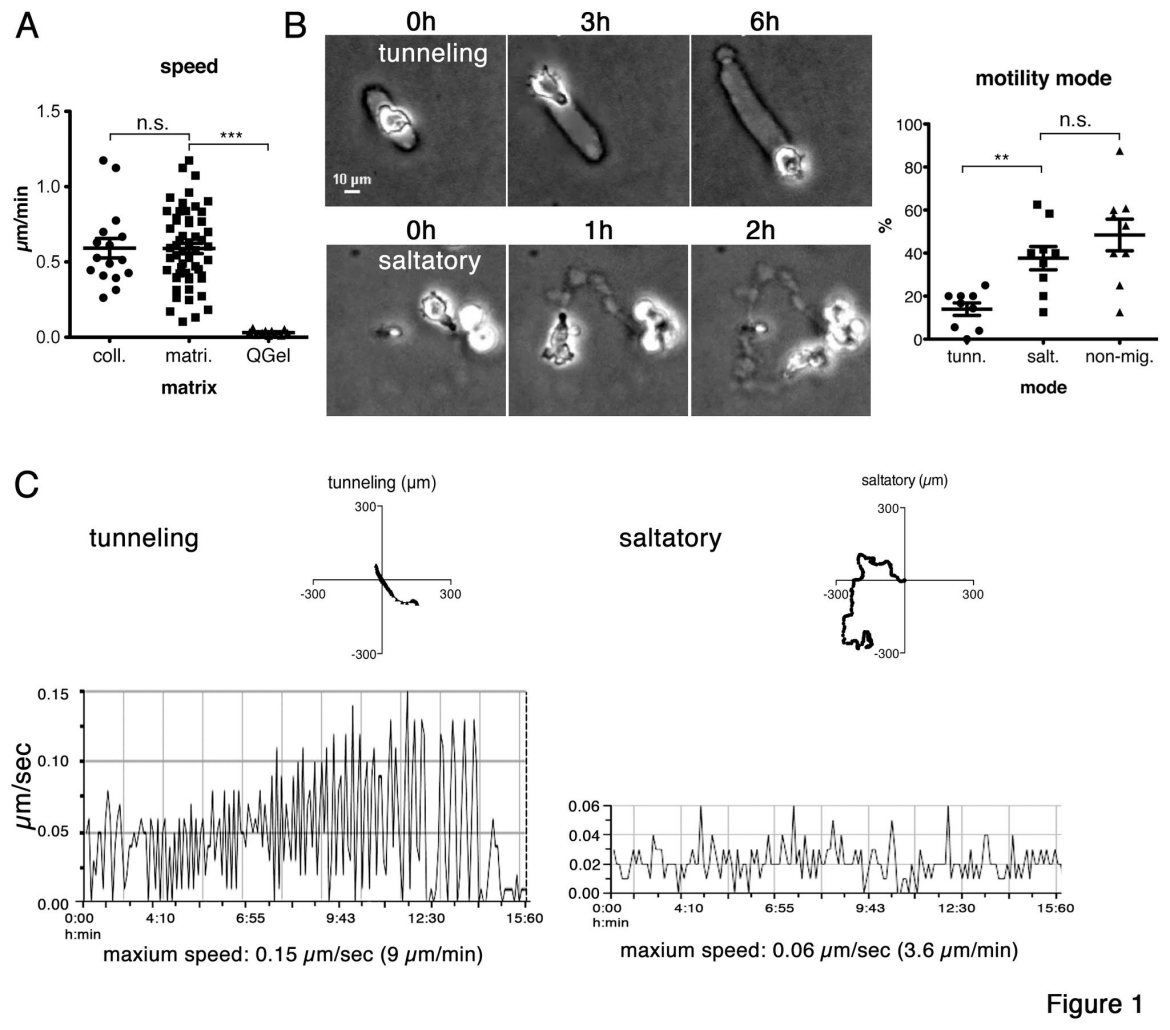
Modes of motility of *T* *. annulata* -**infected** **macrophages** **embed** **in** **matrigel**. (A) Motility of cells embedded in collagen, matrigel or Qgel matrices was recorded for 15 h by live-video microscopy. Vertical scatter dot plots show speed calculated as µm/min (collagen: 55 cells, matrigel: 19 cells, QGel: 10 cells). T-test, *** p < 0.0001. B) Infected cells adopt different modes of motility in matrigel. These include forward movement by tunneling, which requires matrix degradation/engulfment at the leading edge (movie S3 and S5). Alternatively, cells can move in a saltatory mode, where cells squeeze through narrow pores and forward movement oscillates between rapid movements and phases of stagnation (movie S4 and S6). Vertical scatter dot plots show percentage of cells migrating in tunneling (14%) or saltatory (38%) mode. 48% of the cells could not be allocated to one of the two modes (immobile). 147 cells total, T-test, ** p = 0.0014. C) X/Y plots show representative paths of cells migrating in the tunneling (left) or in the saltatory mode (right). Dot plots show representative track lengths of tunneling and saltatory migration in µm. Speed diagrams compare fluctuations in speed development of tunneling and saltatory movements; peak = maximum speed, valley = minimal speed or stagnation.

*T* *. annulata* -infected macrophages migrate efficiently in different 3D environments and they are able to penetrate and migrate in collagen and matrigel matrices. We previously demonstrated that motility of infected cells depends on their capability to polarize [7,29] and to trigger membrane blebs at the leading edge [10]. To better understand morphodynamic characteristics of this rounded/amoeboid cell motility, we visualized and quantified motility of infected cells in collagen and matrigel matrices by time-lapse video microscopy. We first compared speed of TaH12810 cells [10] migrating either in collagen, matrigel or a synthetic matrix based on cross-linked polyethylene glycol (QGel) (Figure 1A). Cells migrated efficiently in both collagen (movie S1) and matrigel (movie S2) while they remained stationary when embedded in QGel matrix (not shown). Interestingly, the comparison of cell speeds in collagen and matrigel revealed no significant differences with an average speed of approximately 0.5 µm/min. Considering the different stiffness and pore sizes of collagen and matrigel, we asked what morphological alterations we could observe at the single cell level in matrigel. Live-cell microscopy revealed two types of motile behavior, which we named tunneling and saltatory. Cells migrating in the tunneling mode, penetrated the matrix rounded with occasionally apparent rear constrictions contrasting the marked central constrictions of cells migrating in the saltatory mode (see below). Tunneling mode cells generated a tunnel-like cavity with a diameter that corresponds to the diameter of the cell (movie S3). In this tunnel, the cells can move at high speed in both directions, simply by switching the polarity axis. Interestingly, degradation and/or engulfment of the matrix occurred mostly on both ends of the tunnel but rarely in an angle off the initial axis of migration. In contrast, cells migrating in the saltatory mode penetrated the matrix in a series of sequential steps that results in a (“saltatory”) forward migration speed that oscillates between stagnation and rapid forward movements (movie S4). These steps are described in more detail below. A significantly larger number of cells (Figure 1B) migrated in the saltatory mode (37.65% ± 5.4), indicating that this form of migration is more favorable when large distances need to be overcome. Consistently, cells migrating in the tunneling mode (13.94% ± 2.89) tended to switch to the saltatory mode (movie S2). We measured the speed (distance/time) of cells migrating in these two different modes and found that cells migrated at maximal speeds when inside a tunnel (from one end to the other) whereby they reached approximately 9 µm/minute (see movie S3). Maximal speeds in the saltatory mode were approximately 3-fold lower with maxima around 3.5 µm/min. Speed diagrams shown in Figure 1C visualize cell displacements in µm from one interval to the next over a time period of 15 hours. The frequency of the oscillation displays the frequency of speed changes over time. In the tunneling mode, the speed is close to zero µm/m (valleys), when the cells hit one end of the tunnel and maximal (peaks) when migrating trough the tunnel. These data show that TaH12810 cells can migrate efficiently in both collagen and matrigel matrices by adopting either a tunneling or a saltatory mode of migration. The cells can migrate at very high speeds in pre-formed matrigel tunnels. However, saltatory migration is more efficient because it results in higher velocity (displacement from origin/time, see dot plots in Figure 1C) and might be used by cells for directional and chemotactic movements. The saltatory mode implies cellular compliance and the capability to expand membranous protrusions into the matrix in order to extend preexisting cavities.

### Actin cytoskeleton dynamics are increased in leading edge protrusions of cells penetrating matrigel

**Figure 2:**
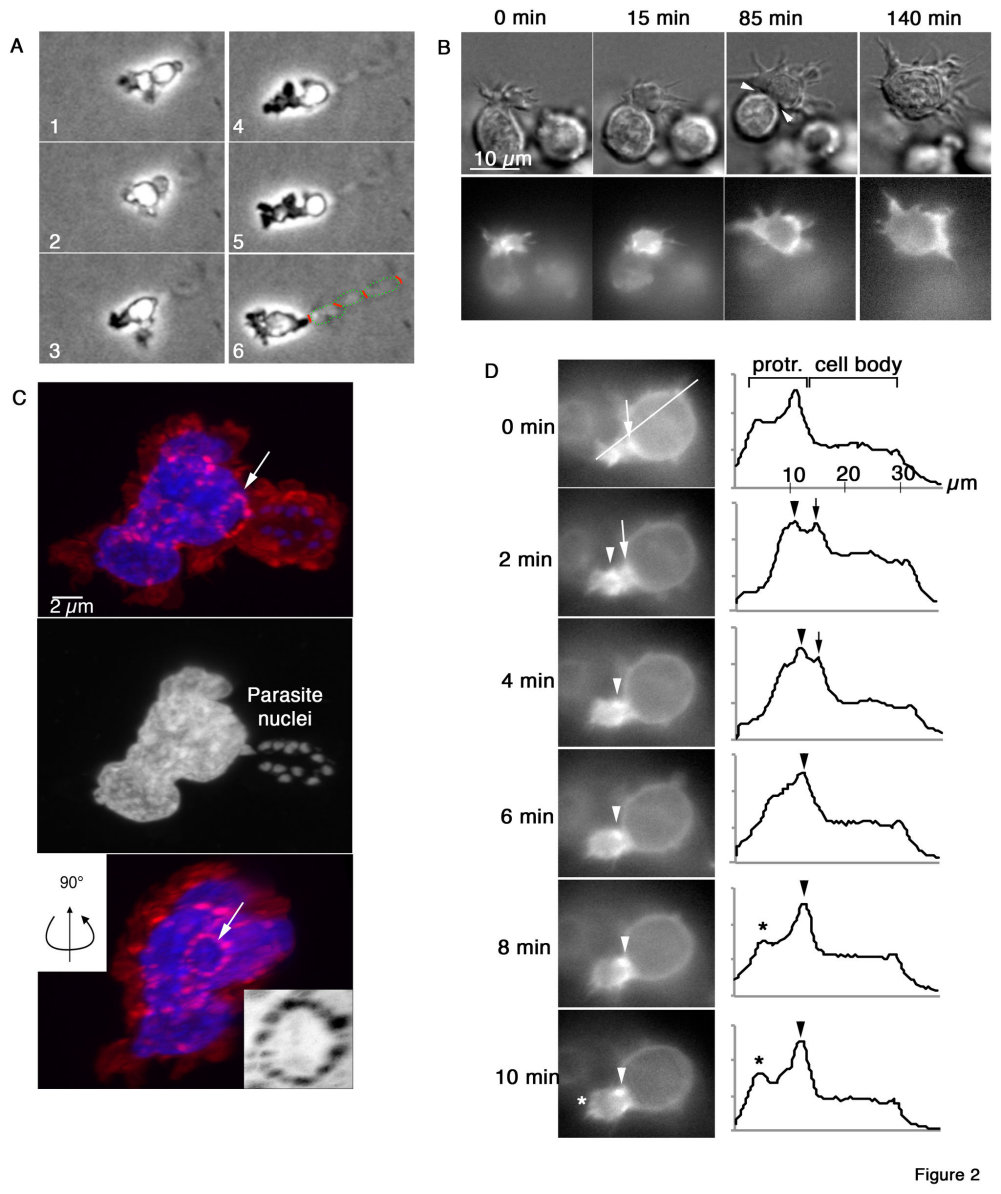
Actin polymerization at invasion front of cells migrating in saltatory mode. **A**) TaH12810 cells were embedded in matrigel and migration was monitored by time-lapse imaging. Green dots indicate circumference of cavities in migration path, red lines indicate position of pores between holes. **B**) Time-lapse microscopy of EGFP-actin expressing TaH12810 cell invading matrigel (movies S7 and S8). Top: grey scale images, bottom: EGFP-actin fluorescence. **C**) 3D reconstruction of confocal microscopy sections of TaH12810 cell migrating in matrigel. Actin cytoskeleton stained with TRITC-phalloidin (red). Arrow indicates actin–rich circle in maximal constriction zone at pore after nuclear translocation (inset shows F-actin in gray scale, 2x magnification). **D**) As in B but focus on initial phase of matrigel invasion. EGFP-actin fluorescence intensity was measured along line indicated and plotted against line length in µm. Arrows indicate peak fluorescence at cell cortex where protrusion emerges, arrowheads F-actin-rich ring at circular constriction zone, asterisks leading edge F-actin.

Closer inspection of migrating cells revealed that cells extended protrusions at the leading edge in the direction of migration and generated pores with a diameter corresponding to approximately 1/4 cell diameter (Figure 2A, movies S6, S7 & S14). Transmigration of the cell body through these pores appeared to be restricted by the compressibility of the nucleus (Figure 2B), which sets the limit for circumferential compression of a cell [30]. In the tunneling mode, cells extended filopodia-like protrusions at the leading edge, while the formation of membrane blebs appeared markedly reduced (Figure S1 and movie S5). During transmigration in the saltatory mode, the cells assumed an hourglass shape, which was hallmarked by a neck zone of maximal compression (Figure 2B, arrowheads). 3D reconstruction of multiple confocal sections revealed that the zone of maximal compression was reinforced inside the cell by an F-actin-rich ring (Figure 2C, arrow). The protrusions at the leading edge were rich in F-actin as well, suggesting that de-novo actin polymerization is required for forward extension of the cell. Indeed, using cells expressing lifeact-GFP, we found that F-actin polymerization was predominately active at the leading edge of invading cells and particularly in protrusions penetrating the matrigel (Figure 2B and movie S8), while polymerization activity was less abundant in the tail. Zones particular rich in F-actin were the submembranous skeletons in the neck zone and near regions at the leading edge where membranous protrusions emerge. To determine what the function of F-actin polymerization at the leading edge might be, we visualized the initial steps of protrusion formation in cells expressing lifeact-GFP. We found that filamentous or sheet-like, actin-rich assemblies resembling lamellipodia of matrix-invading epithelial cell [31], initiated the protrusions. However, instead of assuming spindle shaped morphology like these cells, parasitized macrophages remained rounded and progressively expanded the initial protrusions until large enough to accommodate the nucleus (Figure 2D). Furthermore, we observed accumulation of F-actin at the neck zone and at the leading edge, and overall a markedly increased content of F-actin in the protrusion that clears the way for the cell. Taken together, massive F-actin polymerization at the leading edge follows initial polarization of the cells and is likely needed for invasive migration of these cells.

### ERM protein localization to leading edge, neck and tail of the cells

**Figure 3:**
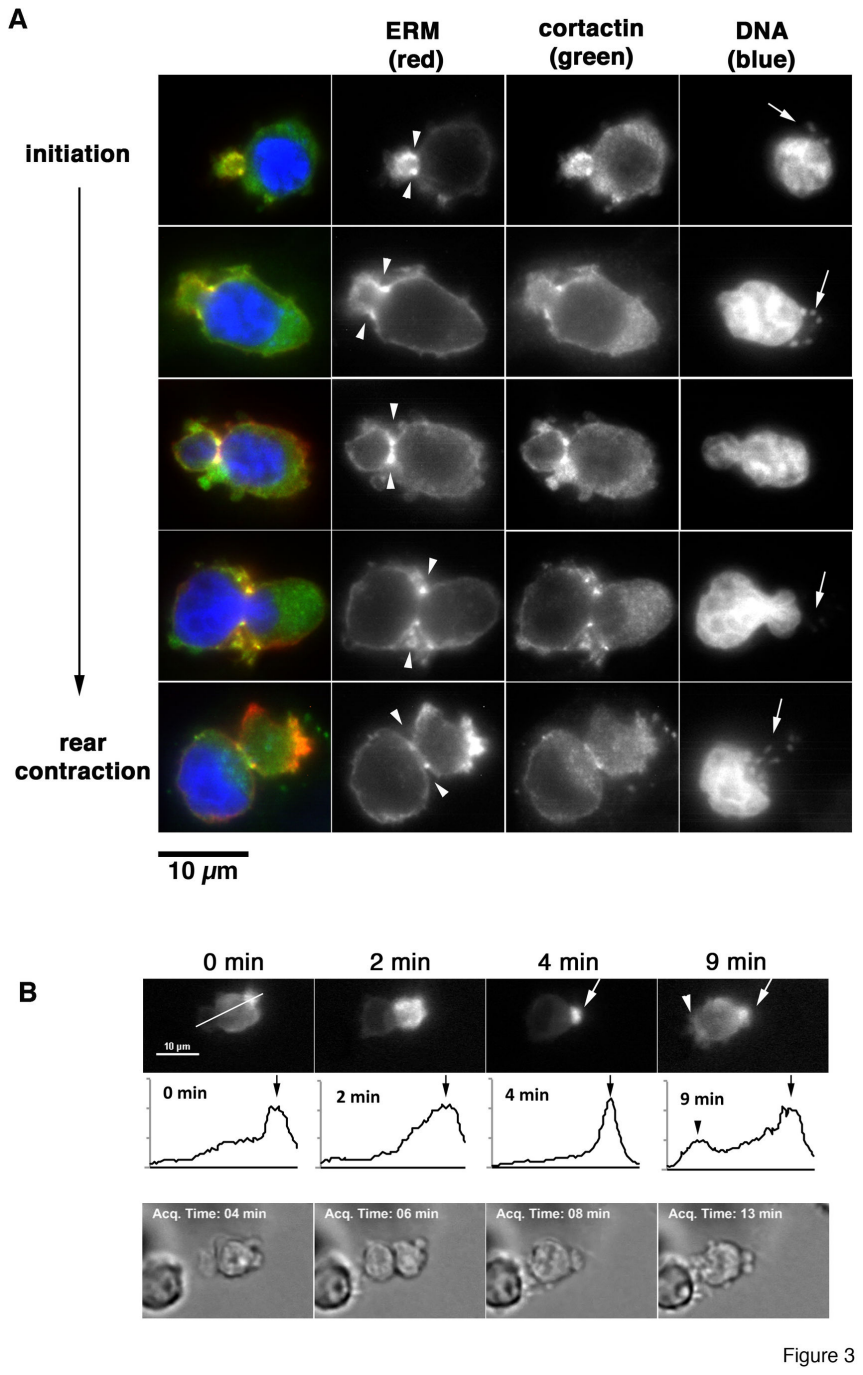
ERM proteins localize sequentially to leading edge, the neck zone and the trailing edge. **A**) If microscopy analysis of ERM protein localization in cells migrating in matrigel using anti-ERM antibodies (red). Leading edge of cells was determined using cortactin localization (green). The location of the trailing edge was confirmed by visualizing host and parasite nuclei (arrows) using hoechst stain. Arrowheads indicate neck zones. **B**) Quantification of YFP-ezrin fluorescence intensity (leading to trailing edge). Top row shows still images of YFP-fluorescence of time lapsed-image acquisition for a period of 9 min. Bottom row are the corresponding gray-scale images. Middle row shows intensity histograms of YFP-fluorescence along the white line (top row left). Arrows indicate tail, arrowhead leading edge.

We have recently described that ERM proteins accumulate in lamellipodia of infected cells migrating on 2D substrata [7]. To determine whether ERM proteins localized to leading edge structures in cells migrating inside 3D matrices as well, we visualized ERM protein distribution at different stages of matrix invasion by immunofluorescence microscopy (Figure 3). We localized the leading edge of migrating *Theileria*-infected macrophages by two ways. First, we localized the parasite by nuclear staining with Hoechst, which visualized both host cell and parasite nuclei. In polarized, migrating cells, the parasite is always located between the host cell nucleus and the trailing edge of the cell (Figure 3A, arrows). Parasite localization with respect to the host cell nucleus can thus be used as a directionality indicator to determine the leading edge. Second, we found that the protein cortactin (Figure S2A and B) and tyrosine phosphorylated proteins (Figure S2C) accumulated at the invasion front in migrating cells. Cortactin is a well-established organizer of the cortical actin cytoskeleton at the leading edge to promote motility and invasiveness of cancer cells [32]. Therefore, we used anti-cortactin immunostaining as leading edge indicator and asked as to whether ERM proteins co-distributed with cortactin. We found that cortactin and ERM protein detection overlapped in invading cells and that co-distribution of the two proteins was particularly evident in the neck zone, where ERM protein accumulation remained prominent throughout most of the penetration process. Once nuclear translocation trough the pore was accomplished, ERM proteins were detected at the rear of migrating cells, suggesting a function of ERM proteins for tail compression and rear retraction. To determine ERM protein distribution in living cells, we expressed YFP-fused ezrin and monitored YFP-ezrin dynamics in cells migrating through matrigel by time-lapse imaging. We detected YFP-ezrin at the plasma membrane near the leading edge and highly enriched in the tail (Figure 3B arrow). After nuclear translocation, YFP-ezrin began to accumulate at the invading leading edge again (Figure 3B arrowhead), which suggested a function of ERM proteins both at leading and trailing edges.

### Host cell MAP4K4 and ERM proteins co-distribute to leading and trailing cell compartments

**Figure 4:**
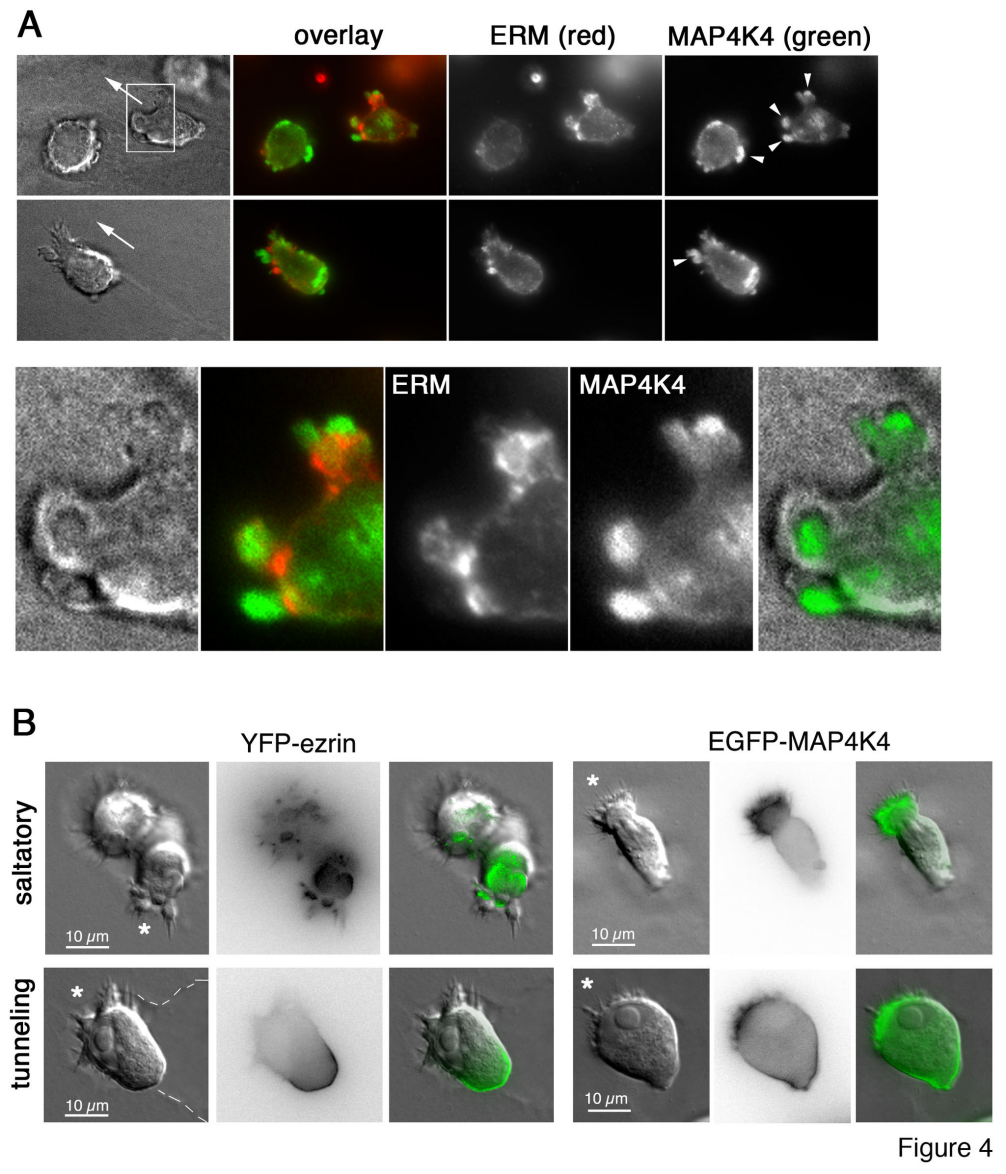
The ERM kinase MAP4K4 localizes to leading and trailing edges of migrating TaH12810 cells. **A**) IF microscopy analysis of ERM and MAP4K4 protein localization in cells migrating in matrigel using anti-ERM and anti-MAP4K4 antibodies. Magnifications are 4x of boxed area and highlight MAP4K4 accumulation in membrane blebs. Arrows indicate direction of migration. **B**) Time-lapse microscopy of TaH12810 cells expressing either YFP-ezrin (movies S11 & S13) or EGFP-MAP4K4 (movies S9 & S10). LA-mCherry was used to visualize leading edge actin dynamics (movie S12). Cells migrate in matrigel either in saltatory (upper) or tunneling (lower) mode (from left to right: grey scale, GFP or YFP fluorescence, overlay). Dotted line indicates cavity boundary, stars indicate leading edge of cells.

ERM protein activity and function are regulated by C-terminal phosphorylation. One C-terminal regulatory kinases is MAP4K4 (mitogen-activated protein kinase kinase kinase kinase 4), a serine/threonine kinase, which phosphorylates ERM proteins for lamellipodia formation [33]. MAP4K4 is an essential kinase for macrophage function in the context of inflammatory signaling induced by TNF-alpha [34], a parasite-induced cytokine expressed in *T* *. annulata* -infected macrophages [22]. If MAP4K4 would be involved in the regulation of ERM function, we would expect it to be localized near leading and trailing edges of invading cells. By IF microscopy, we detected MAP4K4 at the leading edge of matrigel invading cells with apparent polarization (Figure 4A); when differential interference contrast (DIC) images in grey-scale were overlaid with RGB images generated with anti-MAP4K4 staining (Figure 4A magnifications), we observed preferential accumulation of MAP4K4 inside membrane blebs. Interestingly, anti-ERM and anti-MAP4K4 staining did not accurately co-localize; instead, ERM proteins localized predominately to the neck zone and cortex of membrane blebs (Figure 4A, magnification). We confirmed the localization pattern of MAP4K4 and ERM proteins by ectopically expressing MAP4K4 (GFP-MAP4K4) and Ezrin (ezrin-YFP) as fusions to GFP and YFP, respectively. We visualized dynamic alterations in subcellular distribution of the two proteins by fluorescent live-cell imaging in TaH12810 cells migrating inside matrigel. Analogous to the IF data, we detected ectopically expressed MAP4K4 predominately at the leading edge of migrating cells (Figure 4B, movies S9 & S10). However, we also observed GFP fluorescence in the trailing end of the cells (Figure 4B, lower), which suggested that MAP4K4 could be distributed to the rear and needed there as well. We observed strong ezrin-YFP fluorescence in trailing edge membranes and somewhat weaker in protrusions at the leading edge (Figure 4B, lower, movie S13), where F-actin dynamics are particularly high (Figure 4B, lower, movie S12; see movie S11 for grey scale live-cell imaging of same cell). Furthermore, ezrin-YFP accumulated in the neck zone during matrix penetration, where it remained throughout the penetration process. Combined, these observations indicate that spatially restricted accumulation of ERM proteins and of their regulator MAP4K4 could contribute to leading edge actin and membrane dynamics in *T* *. annulata* -infected cells.

### Filopodia-like protrusions lead the way while membrane blebbing expands the matrix for invading cells

**Figure 5:**
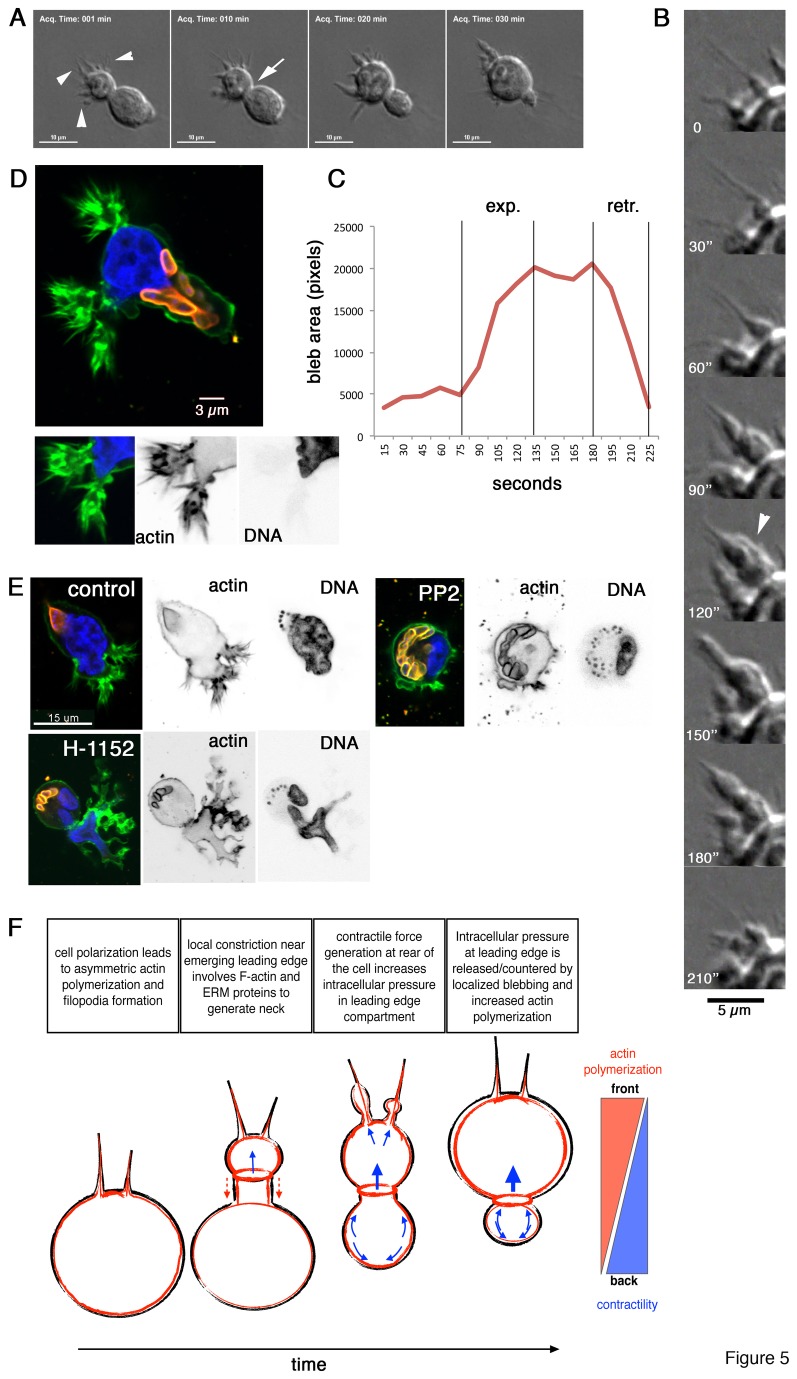
Membrane blebs evolve along filopodia-like protrusions at leading edge. **A**) Still images of DIC live-cell microscopy (movie S14). Arrowheads indicate filopodia-like protrusions, arrow neck zone of maximal compression. **B**) Higher magnification of leading edge of cell shown in A. Arrowhead highlights blebbing protrusion at maximal expansion. **C**) Quantification of surface area of blebbing protrusion shown in B. **D**) Confocal microscopy image of TaH128101 cells invading matrigel. Green: F-actin, red: parasite surface (anti-TaSP), blue: DNA. Insets show leading F-actin and host cell nuclear DNA. **E**) as D but cells were incubated in the presence of either the Src kinase inhibitor PP2 (5 µM) or the Rho kinase inhibitor H-1152 (5 µM). **F**) model of F-actin distribution and leading edge membrane dynamics of TaH12810 cells invading matrigel.

To further understand dynamic alterations at the leading edge during matrigel invasion, we monitored the invasion process at high spatial and temporal resolution. Figure 5A shows how a cell migrates in saltatory mode, thereby penetrating the matrix and translocating the nucleus in a stepwise process. Figure 5B focuses on the invasion zone at the very leading edge of the same cell and shows the dynamic morphological alterations in that zone. We observed that membrane blebs expand along existing filopodia-like protrusions, whereby expansion began at the base of the protrusion and extended outward. We quantified the expansion and retraction processes by monitoring and measuring the size of a growing bleb every 15 seconds for 3 minutes (Figure 5B and C). Unlike membrane blebs of TaH12810 cells embedded in collagen (movie S1), which showed rapid bleb expansion and slow retraction [10], expansion and retraction dynamics of leading edge blebs in cells embedded in matrigel were comparable (Figure 5C). Confocal microscopy analysis of the blebbing protrusions showed that they consisted of rounded expansions that were lined with F-actin and decorated with multiple filopodia (Figure 5D). We have previously shown that Src kinase activity is needed for the polarization of *Theileria*-infected macrophage and herein we detected increased protein tyrosine phosphorylation at the leading edge of invading cells (Figure S2). To determine whether Src kinase activity could be a determinant for TaH12810 morphology during invasion, we treated infected cells embedded in matrigel with the Src kinase inhibitor Su6656 (not shown) or PP2 (Figure 5E). Both inhibitors reduced asymmetric, cortical F-actin accumulation and the formation of invasion structures compared to control cells. In contrast, cell treated with the Rho kinase inhibitor H-1152 displayed polarized morphology and massive protrusions (Figure 5E). However, analogous to what we observed in collagen-embedded TaH12810 cells [10], these protrusions appeared disorganized and the nucleus failed to transmigrate. 5F schematically shows dynamic morphological alterations during matrigel invasion, bleb formation, matrix expansion and retraction of the trailing edge.

## Discussion

In this study we visualized dynamic morphological alterations of rounded/amoeboid cell invasion by live and fixed cell microscopy and sought to determine molecules associated with relevant invasion structures. We used bovine macrophages infected and oncogenically transformed by the protozoan parasite *Theileria annulata* as a model for rounded/amoeboid cell motility, which we found to be highly motile and invasive when embedded in 3D matrices. We show that the cells can switch motility modes in matrigel and either migrate in a tunneling or a saltatory mode. Cells migrating in the tunneling mode displayed reduced membrane blebbing at the leading edge and appeared to depend on matrix degradation and/or engulfment. In contrast, cells migrating in the saltatory mode depended on progressive matrix compression, contractility and nuclear deformation. A characteristic feature of cells migrating in the saltatory mode is the conjunction of filopodia-like protrusions with membrane blebbing at the leading edge, which we found herein to be a highly efficient mechanism to penetrate stiff matrices. By combining these seemingly unrelated cellular protrusions structures in an invasion machinery, cells can efficiently overcome physical barriers by locally directed matrix expansion and subsequent transmigration through pores markedly smaller that the cell diameter. Our study thus highlights a novel aspect of the plasticity of eukaryotic cell motility, which likely determines the motile capabilities of activated myeloid cells or invasive cancer cells.

We propose that asymmetrical rigidification of the cortical cytoskeleton at the rear increases contractility and intracellular pressure. This increased intracellular pressure is relieved by membrane expansion at sites of reduced cortical stiffness. Such sites include zones of high actin dynamics, where the cortical cytoskeleton and the plasma membrane transiently detach [35], such as those at the leading edge. Indeed, we observed membrane blebs that develop and decay along leading edge filopodia-like protrusions, which is indicative for enhanced intracellular pressure. Rho kinase is an essential component for blebbing motility of cancer cells [19] and inhibition of Rho kinase activity impairs TaH12810 migration inside 3D matrices (Figure 5E and [10]). However, Rho kinase activity is dispensable for the formation of leading edge protrusion in TaH12810 cells as these can still form in the presence of Rho-kinase inhibitor H-1152 (Figure 5E). Therefore, matrix invasion in transformed macrophages is mechanistically analogous to invading breast cancer cells where Rho-kinase-MLC-driven contractility is dispensable for protrusion formation but required for forward movement of the cell body [19]. Unfortunately, we were not able to determine Rho GTPase activity in infected cells because the amount of Rhotekin-bound bovine Rho was below the detection limit of our assay. Interestingly however, we detected markedly higher Cdc42 activity in the virulent TaH12810 cells compared to the culture-attenuated Thei cell line or drug-cured Thei cells (Figure S3, see also below).

We observed ezrin distribution in living, invading cells both at the leading and the trailing edges, where it co-distributes with its potential activator MAP4K4 [33]. Activated ERM proteins stabilize the cortical actin cytoskeleton and adjacent membranes by tethering F-actin to transmembrane receptors [36] and the expression of dominant active T567D ezrin reduces membrane bleb formation [37]. Conversely, presence of ezrin and moesin was evidenced in expanding and retracting membrane blebs [38], suggesting that their reversible phosphorylation might determine ERM effects on bleb formation. Since MAP4K4 and ERM proteins co-distribute to the leading edge of invading cells, it is conceivable that MAP4K4-mediated ERM phosphorylation is needed to balance cortical rigidity and local membrane dynamics. However, several other kinases including PKC [39], LOK [40] and ROCK [41] have been shown to phosphorylate and activate ERM proteins. Therefore, additional studies are needed to determine the possible functional interaction of MAP4K4 and ERM proteins and their spatio-temporal activation patterns in *Theileria*-transformed macrophages.

Our data contrast with the motility mode of human macrophages and fibrosarcoma cells [14], where the matrix architecture (density and rigidity of the matrix) influenced the motility of both normal macrophages and cancer cells: In low density, fibrillar matrix (fibrillar collagen), the cells migrate in a rounded/amoeboid mode, whereas in high density and stiff matrices (matrigel or gelled collagen), the cells adopt a spindle-like, proteolytic mode. We tested whether the elimination of the parasite reverted amoeboid motility in matrigel and promoted spindle morphology. However, only very few cured (drug-treated cells) cells free of parasites survived when embedded in matrigel (not shown). We do not yet understand what the underlying cause of decreased survival of cured cells in matrigel is but it is possible that reduced autocrine stimulation of isolated cells in a stiff matrix environment is not sufficient for cured cells to survive. In human cancer cells, filopodia-like protrusions are needed to promote survival and proliferation of extravasating cancer cells by inducing ß1-integrin-dependent activation of the focal adhesion kinase FAK. Consistent with the possibility that filopodia-like protrusions are constitutively induced and important for infected cell motility and survival is our observation of high Cdc42 GTPase activity in TaH12810 cells and of its inactivation upon parasite elimination (Figure S3). One possible mechanism of Cdc42 induction in *Theileria*-transformed cells could involve TNF-alpha, which is induced in TaH12810 cells in parasite-dependent manner [22] and was shown to promote robust filopodia induction via Cdc42 activation in mouse embryonic fibroblasts [42].

The present study leaves several issues unresolved, namely what the molecular basis of TaH12819 cells is that determines whether the cells migrate either in the tunneling or the saltatory mode, what controls ERM protein localization to the leading edge and how initial polarization is triggered. However, it reveals for the first time that a divergent form of membrane blebs can develop in conjunction with filopodia-like fibrillar protrusions at the leading edge, which combined may facilitate matrix invasion of rounded/amoeboid migrating cells.

## Supporting Information

Figure S1 **Cells migrating in tunneling and those migrating in saltatory mode are morphologically distinguishable.** Cells were embedded in matrigel. Still images of time-lased image acquisition for 50 min are shown 24h after seeding (movies S5 & S6). White dotted lines indicate boundaries of differentially shaped cavities formed by the cells.
(TIF)

Figure S2 **Determining direction of migration by cortactin localization.** **A**) Cortactin accumulates at leading edge of matrix-invading cell. Cortactin and ERM proteins were visualized in matrigel embedded cells by fluorescence microscopy. DIC image (left) shows cluster of cells with polarized single cell (a) migrating away from cluster (b). Arrow indicates direction of migration. Cortactin (green) and ERM accumulate near the leading edge in migrating cell. a: polarized, matrix-invading cell; b: non-polarized, stationary cell. Magnifications: 4x. **B**) 3D reconstruction of confocal sections of a TaH12810 cell migrating in collagen. Top-left image shows cell from top. Top-right shows cell after counter-clockwise horizontal rotation by 45°. Bottom-right as top-left but without green (actin) fluorescence. Bottom-left shows schematic interpretation of microscopy images. Staining: green: actin (phalloidin A4588), red: parasite surface (anti-TaSP, Cy3), blue: cortactin (anti-cortatcin, Cy5), white: DNA (hoechst). **D**) Fluorescence microscopy analysis of matrigel embedded TaH12810 cells. Staining: green: pTyr (anti-pTyr, A4588), red: ERM proteins (anti-ERM, TRITC), blue: DNA (hoechst). Arrows indicate direction of migration. **C**) Schematic illustration of infected cell migrating in matrigel.
(TIF)

Figure S3 **Cdc42 activity is increased in virulent TaH12810 cells.** Rho, Rac and Cdc42-pull down assay from *T* *. annulata* -infected and BW720c-cured macrophages. Western-blots of GTP-bound active (upper) and total GTPases (lower) using anti-Rho, anti-Rac and anti-Cdc42 antibodies as indicated are shown.
(TIF)

Movie S1 **TaH12810 cell embedded in collagen 24h.** Recording time: 45 min, intervals: 4.5 sec, run time: 18 sec, FPS: 33.
(M4V)

Movie S2 **TaH12810 cells embedded in matrigel.** Recording time: 1200 min, intervals: 300 sec, run time: 24 sec, FPS: 30.
(M4V)

Movie S3 **TaH12810 cells embedded in matrigel.** Recording time: 930 min, intervals: 30 sec, runtime: 18 sec, FPS: 30.
(M4V)

Movie S4 **TaH12810 cells embedded in matrigel.** Recording time: 1200 min, intervals: 30 sec, run time: 24 sec, FPS: 30.
(M4V)

Movie S5 **TaH12810 cells embedded in matrigel.** Recording time: 55 min, intervals: 15 sec, run time: 21 sec, FPS: 30.
(M4V)

Movie S6 **TaH12810 cells embedded in matrigel.** Recording time: 53 min, intervals: 15 sec, run time: 17 sec, FPS: 30.
(M4V)

Movie S7 **TaH12810 cells embedded in matrigel.** Recording time: 300 min, intervals: 60 sec, run time: 18 sec, FPS: 30.
(M4V)

Movie S8 **TaH12810 cells embedded in matrigel.** Actin-GFP fluorescence, Recording time: 300 min, intervals: 60 sec, run time: 18 sec, FPS: 30.
(M4V)

Movie S9 **TaH12810 cells embedded in matrigel.** DIC imaging, Recording time: 180 min, intervals: 6 sec, run time: 26 sec, FPS: 30.
(M4V)

Movie S10 **TaH12810 cells embedded in matrigel.** Actin-GFP fluorescence, Recording time: 180 min, intervals: 6 sec, run time: 26 sec, FPS: 30.
(M4V)

Movie S11 **TaH12810 cells embedded in matrigel.** Phase-contrast, Recording time: 266 min, intervals: 60 sec, run time: 9 sec, FPS: 30.
(M4V)

Movie S12 **TaH12810 cells embedded in matrigel.** Lifeact-cherry fluorescence, Recording time: 266 min, intervals: 60 sec, run time: 9 sec, FPS: 30.
(M4V)

Movie S13 **TaH12810 cells embedded in matrigel.** Ezrin-YFP imaging, Recording time: 266 min, intervals: 60 sec, run time: 9 sec, FPS: 30.
(M4V)

Movie S14 **TaH12810 cells embedded in matrigel.** DIC imaging, Recording time: 50 min, intervals: 15 sec, run time: 19 sec, FPS: 30.
(M4V)
