# Supplementary figures and images for: Filopodia and Membrane Blebs Drive Efficient Matrix Invasion of Macrophages Transformed by the Intracellular Parasite Theileria annulata
Source: PLoS One. 2013 Sep 24;8(9):e75577. doi: 10.1371/journal.pone.0075577 (PMC3782453; doi:10.1371/journal.pone.0075577)

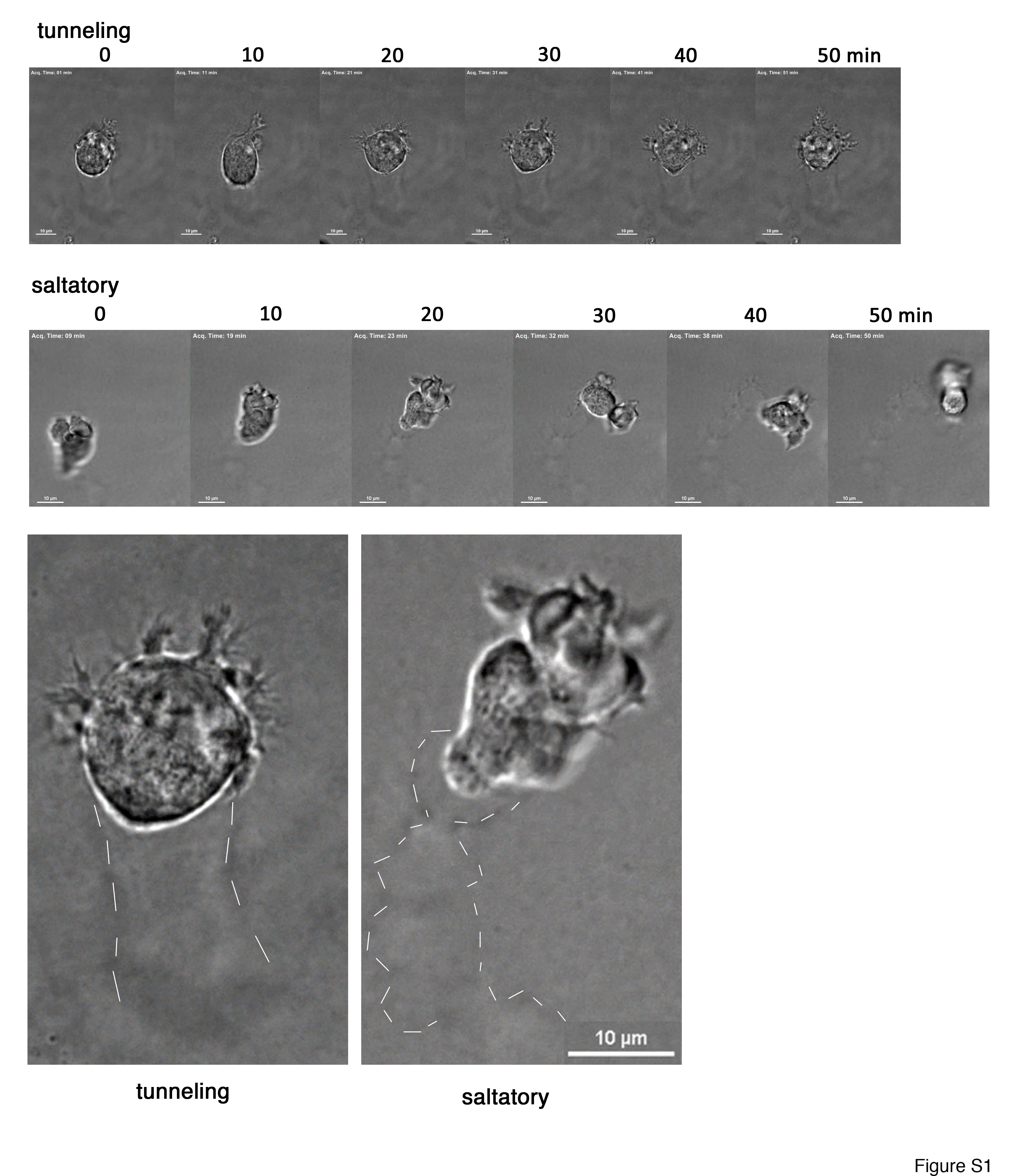

Supplement: Figure S1 — Cells migrating in tunneling and those migrating in saltatory mode are morphologically distinguishable. Cells were embedded in matrigel. Still images of time-lased image acquisition for 50 min are shown 24h after seeding (movies S5 & S6). White dotted lines indicate boundaries of differentially shaped cavities formed by the cells. (TIF) [file pone.0075577.s001.tif]

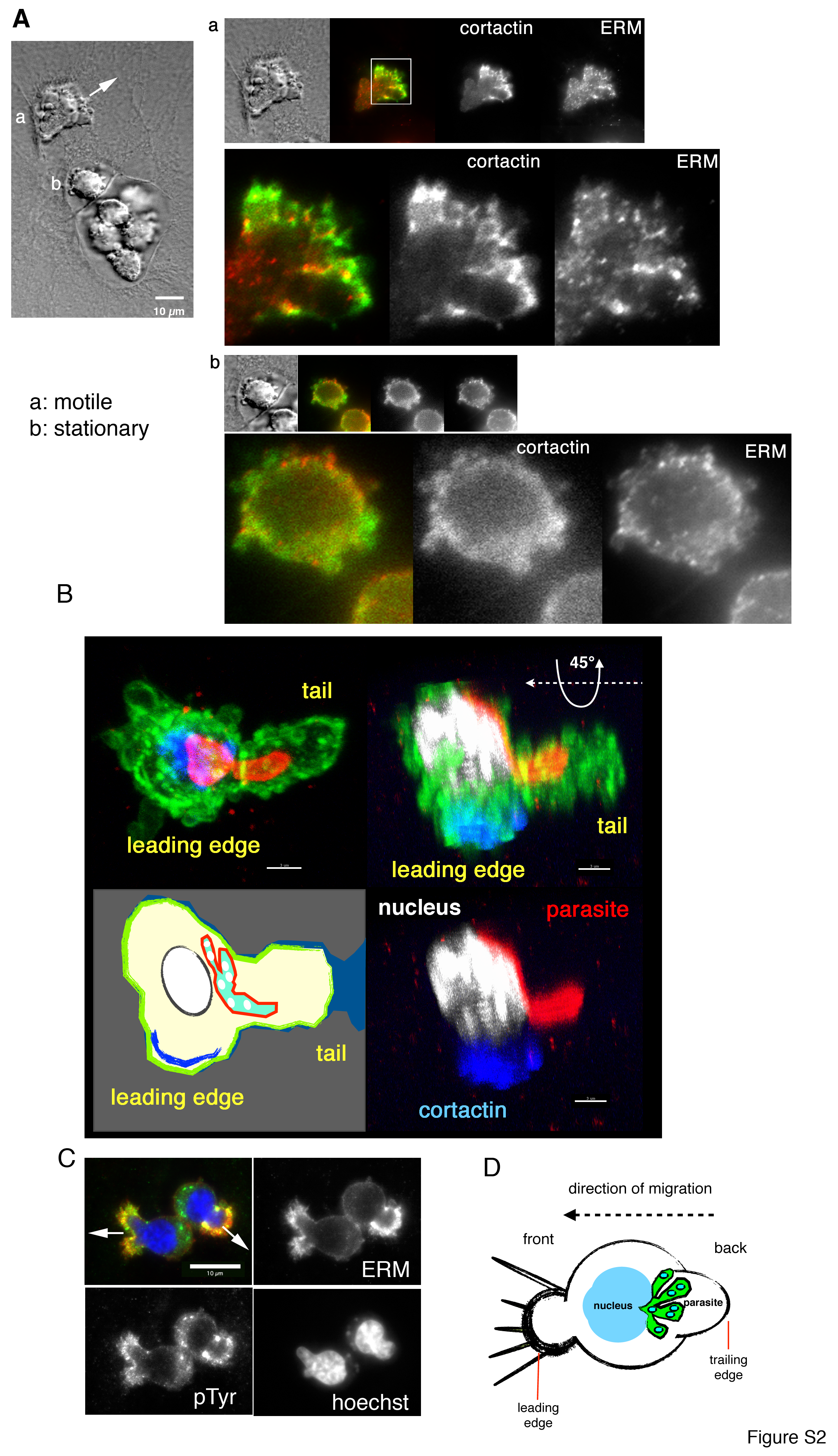

Supplement: Figure S2 — Determining direction of migration by cortactin localization. A) Cortactin accumulates at leading edge of matrix-invading cell. Cortactin and ERM proteins were visualized in matrigel embedded cells by fluorescence microscopy. DIC image (left) shows cluster of cells with polarized single cell (a) migrating away from cluster (b). Arrow indicates direction of migration. Cortactin (green) and ERM accumulate near the leading edge in migrating cell. a: polarized, matrix-invading cell; b: non-polarized, stationary cell. Magnifications: 4x. B) 3D reconstruction of confocal sections of a TaH12810 cell migrating in collagen. Top-left image shows cell from top. Top-right shows cell after counter-clockwise horizontal rotation by 45°. Bottom-right as top-left but without green (actin) fluorescence. Bottom-left shows schematic interpretation of microscopy images. Staining: green: actin (phalloidin A4588), red: parasite surface (anti-TaSP, Cy3), blue: cortactin (anti-cortatcin, Cy5), white: DNA (hoechst). D) Fluorescence microscopy analysis of matrigel embedded TaH12810 cells. Staining: green: pTyr (anti-pTyr, A4588), red: ERM proteins (anti-ERM, TRITC), blue: DNA (hoechst). Arrows indicate direction of migration. C) Schematic illustration of infected cell migrating in matrigel. (TIF) [file pone.0075577.s002.tif]

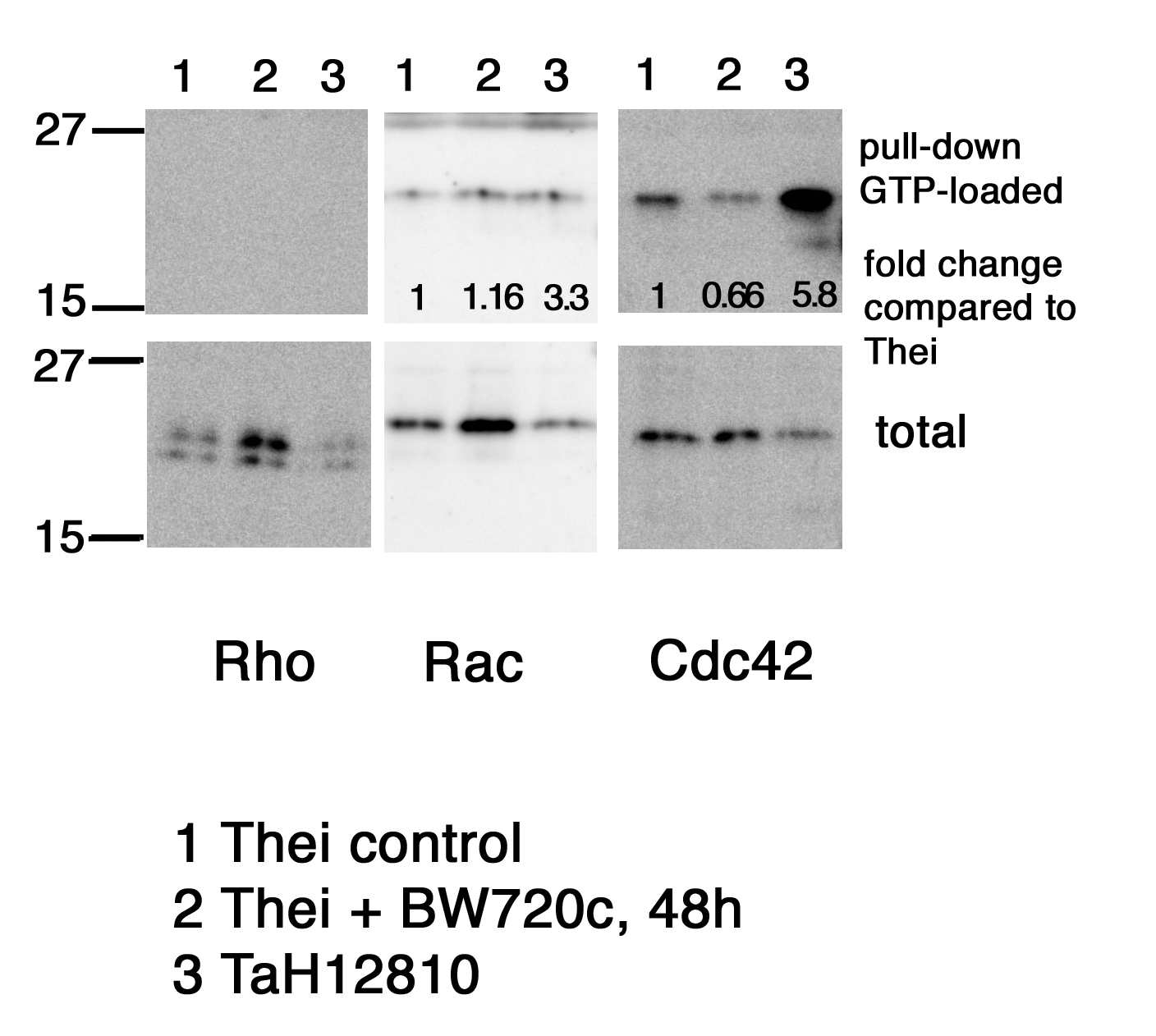

Supplement: Figure S3 — Cdc42 activity is increased in virulent TaH12810 cells. Rho, Rac and Cdc42-pull down assay from T . annulata -infected and BW720c-cured macrophages. Western-blots of GTP-bound active (upper) and total GTPases (lower) using anti-Rho, anti-Rac and anti-Cdc42 antibodies as indicated are shown. (TIF) [file pone.0075577.s003.tif]
